# Brazilian Dialysis Survey 2023

**DOI:** 10.1590/2175-8239-JBN-2024-0081en

**Published:** 2025-01-27

**Authors:** Fabiana B. Nerbass, Helbert do Nascimento Lima, Jorge P. Strogoff-de-Matos, Bruno Zawadzki, José A. Moura-Neto, Jocemir Ronaldo Lugon, Ricardo Sesso

**Affiliations:** 1 Fundação Pró-Rim, Joinville, SC, Brazil.; 2 Universidade da Região de Joinville, Joinville, SC, Brazil.; 3 Fresenius Medical Care, Rio de Janeiro, Brazil.; 4 DaVita Tratamento Renal, Rio de Janeiro, RJ, Brazil.; 5 Escola Bahiana de Medicina e Saúde Pública, Salvador, BA, Brazil.; 6 Universidade Federal Fluminense, Niterói, RJ, Brazil.; 7 Universidade Federal de São Paulo, São Paulo, SP, Brazil.

**Keywords:** Hemodialysis, Peritoneal Dialysis, Epidemiology

## Abstract

**Introduction:**

The annual Brazilian Dialysis Survey (BDS) supports and contributes to the development of national health policies. Objective: To report the 2023 epidemiological data from the BDS of the Brazilian Society of Nephrology (BSN).

**Methods:**

A survey was carried out in a voluntary sample of Brazilian chronic dialysis centers using an online questionnaire covering clinical and epidemiological aspects of patients on chronic dialysis, and characteristics of dialysis centers. For estimates of prevalence, incidence, and source of payment for dialysis, data were obtained from a random sample of dialysis centers.

**Results:**

A total of 37.5% (n = 332) of the centers voluntarily responded to the online questionnaire, and 124 were randomly selected for specific estimates of prevalence and incidence rates. It was estimated that on July 1, 2023, the total number of patients on dialysis was 157,357 and 51,153 started dialysis in 2023. The estimated prevalence and incidence rates of patients per million population (pmp) were 771 and 251, respectively. Of the prevalent patients, 88.2% were on hemodialysis, 8.0% on hemodialfiltration, and 3.8% on peritoneal dialysis. The prevalence of anemia (Hb <10g/dL) was 29% and hyperphosphatemia (P >5.5mg/dL), 30%. There was an increase in the frequency of use of cinacalcet and paricalcitol. The estimated overall crude annual mortality rate was 16.2%.

**Conclusions:**

Estimates from a random sample of dialysis facilities show that the absolute number and the prevalence rate of patients on chronic dialysis continue to increase. A growing number of patients were undergoing hemodiafiltration and using cinacalcet and paricalcitol for hyperparathyroidism treatment.

## Introduction

Since 1999, the Brazilian Society of Nephrology (BSN) has promoted the Brazilian Dialysis Survey (BDS), a national online survey to collect and analyze epidemiological and clinical aspects of patients undergoing chronic dialysis. This initiative has provided relevant information for developing health policies and strategies to improve the care of individuals undergoing chronic dialysis treatment in the country.

Previous surveys used only samples of voluntary participants, which may have made the results more susceptible to selection bias. In this report, some data were obtained from a voluntary survey, and for the first time, data were also obtained from a random sample of dialysis facilities across the country to enhance the accuracy of the estimates for prevalence rates, incidence rates, and payment source for dialysis.

This article reports on the main results of the 2023 BDS and compares them with previous surveys.

## Methods

### Prevalence, Incidence, and Source of Payment For Dialysis

For estimates of prevalence, incidence, and source payment for dialysis, data were obtained through a random sampling of dialysis centers. From a list of dialysis centers in each geographic region (North, Northeast, Central-West, Southeast, and South), centers were selected through a sequential random sample method. The initial dialysis unit was selected from a table of random numbers, and subsequent units followed a fixed sequence of repetitions. Approximately 20% of dialysis centers were selected in each geographic region, except for the Southeast, which has a total of 450 active centers, from which a sample of 15% of centers was chosen. An additional 50% of the selected centers was included to compansate for nonresponse. A minimum target of 70% of the planned number of dialysis facilities was established.

Initially, the selected dialysis units were contacted via e-mail to inform the following data: 1. Total number of patients on chronic dialysis on July 1^st^, 2023; 2. Number of patients with dialysis funding by private healthcare insurance on July 1^st^, 2023; and 3. Number of patients who started chronic dialysis during July 2023. To reinforce the percentage of respondents, a subsequent telephone contact was made with the units that did not answer the electronic messages.

To calculate the estimates, the country’s regions (North, Northeast, Central-West, Southeast, and South) were considered as strata, and it was noticed that sample sharing between the strata was not proportional. As a result, weights were introduced in the data analysis stage to compensate for variations in sampling probabilities and restore proportionality (Table 1). As the response rates varied by region, the weights were adjusted to allow the distribution of the sample across the strata to that of the study population. Given the sampling method employed, simple stratified sampling, the average number of dialysis patients per center was estimated on July 1^st^, and the average number of new patients in the year for each region of the country, with the respective 95% confidence intervals (95% CI), was calculated. The results were then expanded to the total number of units in each region. The percentage of patients financed by public and private healthcare insurance was obtained through simple proportion in the drawn sample.

**Table 1:** Sampling procedures in dialysis facilities across the country, sampling fractions, design weights, response rates, and adjusted weights by region

Region	Facilities			Sampling fraction	Design weight	Response rate	Adjusted weight
	Existing	Sampled	Responding				
North	54	18	10	0.333	3.003	0.556	5.401
Northeast	167	51	27	0.305	3.279	0.529	6.198
Central-West	87	27	17	0.310	3.226	0.630	5.120
Southeast	421	95	40	0.225	4.444	0.421	10.555
South	157	48	30	0.305	3.278	0.625	5.244
Total	886	239	124			0.5188	

### Data from the Voluntary Survey

People designated by the responding dialysis centers filled out an online questionnaire on the BSN website. It contained queries about the sociodemographic, clinical, and therapeutic parameters of patients on chronic dialysis and was available from August to November 2023. Participation in the survey was voluntary, and all dialysis centers registered at BSN were invited to participate by e-mail as well as BSN media. After the initial invitation, new reminders were sent monthly to centers that had not yet provided their data, and direct contact was made with BSN regional presidents and managers of international corporations offering dialysis services in Brazil.

The data for each center were collected in aggregate form, rather than individually (prevalence of predetermined characteristics was questioned, such as the number of patients on hemodialysis, the number of patients with diabetic kidney disease, the number of male patients, and other characteristics related to renal replacement therapy). The annual mortality was estimated from events in July 2023. To calculate the prevalence and incidence rates, we used national and regional population data from the Brazilian Institute of Geography and Statistics (IBGE): total population of the 2022 census plus the mean annual growth rate of 0.52%^1^.

Most data were descriptive and the results were compared with data from previous years.

Estimated total annual number of deaths was obtained from: the number of deaths reported in July multiplied by 12, divided by the proportion of active participating centers.

Estimated crude annual mortality rate (%) was obtained from: the estimated total number of deaths in 2022 multipled by 100, divided by the estimated number of dialysis patients on July 1^st^.

## Results

In July 2023, 886 active chronic dialysis centers were registered at BSN, 1.6% higher than in 2022. In the whole country, there were 4.3 dialysis centers per million population (pmp), with lower rates in the Northeast (3.0 pmp) and North (3.1 pmp) regions compared with the Southeast (4.9 pmp), Central-West (5.3 pmp), and South (5.2 pmp) regions.

### Random Sample Estimates

#### Estimated Incidence and Prevalence Rates from the Random Sample

Fourteen percent of the active Brazilian dialysis centers participated in the random sample (124/886), 18% in the North (10/54), 16% in the Northeast (27/167), 19% in the Central-West (17/87), 9% in the Southeast (40/421), and 19% in the South (30/157) centers.

**Figure 1:**
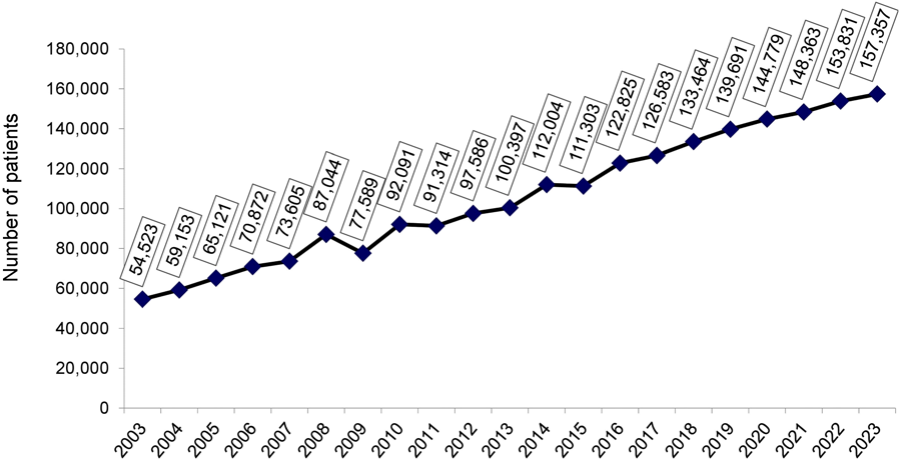
Estimated number of patients on chronic dialysis per year.

**Figure 2:**
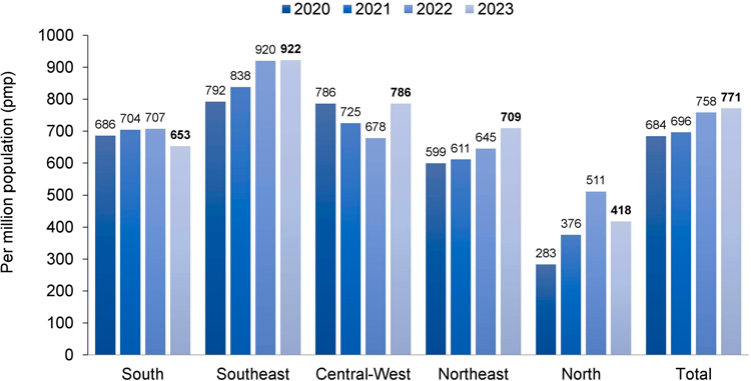
Estimated prevalence rate of patients on dialysis by geographic region in Brazil, per million population.

The estimated total number of patients on July 1^st^, 2023 was 157,357 (95% CI 139,164–175,153), 2.3% higher than in July 2022, confirming the increasing trend in the number of patients on dialysis observed in recent years (Figure 1). The prevalence rate of dialysis patients also continued to rise, from 758 pmp in 2022 to 771 pmp (95% CI 682-858 pmp) in 2023. When examining this indicator by region, rates decreased in the North and the South (Figure 2). The estimated number of new dialysis patients in 2023 was 51,153. The overall incidence rate was 251 pmp (95% CI 213–288 pmp), higher than in 2022 when it reached 214 pmp, ranging from 137 pmp in the North to 311 pmp in the Central-West.

The distribution of patients according to funding sources observed in the random sample survey was 75.6% financed by the public health system and 24.4% by private health insurance. Public funding varied across regions, with higher participation in the Northeast (84%), followed by the South (81%), North (76%), Southeast (70%), and Central-West (64%).

### Voluntary Survey

There were 332 (37.5%) participating centers in the voluntary survey, a higher percentage than in the last three years, which was around 30%. When considering the response rate by the number of dialysis centers per region, the region with the highest participation was the Central-West (46%), followed by the Northeast (41%), South (39%), Southeast (34%), and North (33%). The number of patients in the current BDS was 46% higher than in 2022 (62,617 vs. 42,868).

**Figure 3:**
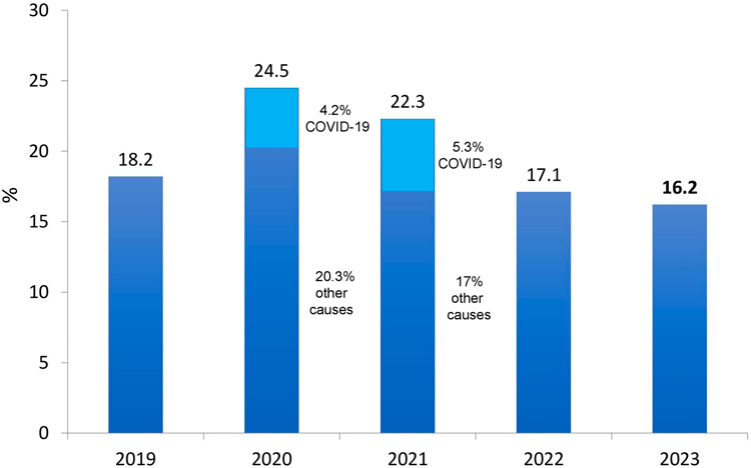
Estimated annual crude mortality rate of dialysis patients.

The estimated number of deaths in the intire year was 27,142. The annual crude mortality rate decreased slightly from 17.1% in 2022 to 16.2% in 2023 (Figure 3).

### Demographic and Clinical Characteristics

**Figure 4:**
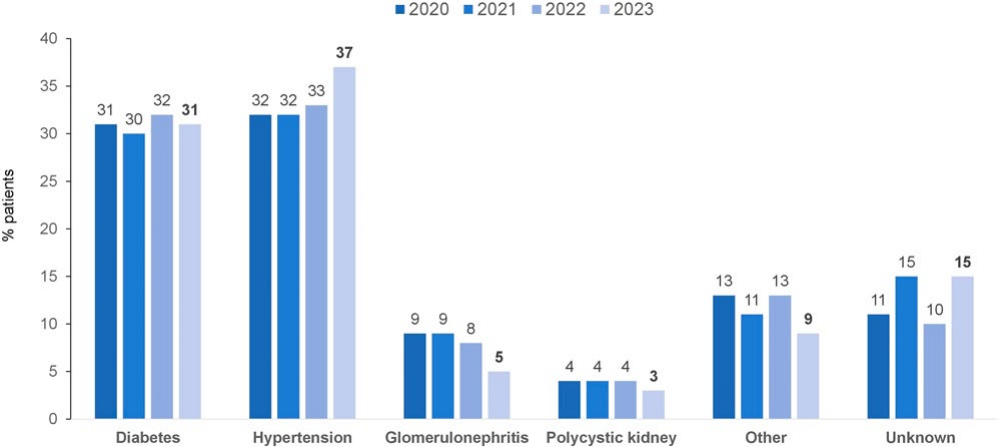
Distribution of dialysis patients according to chronic kidney disease etiology.

**Figure 5:**
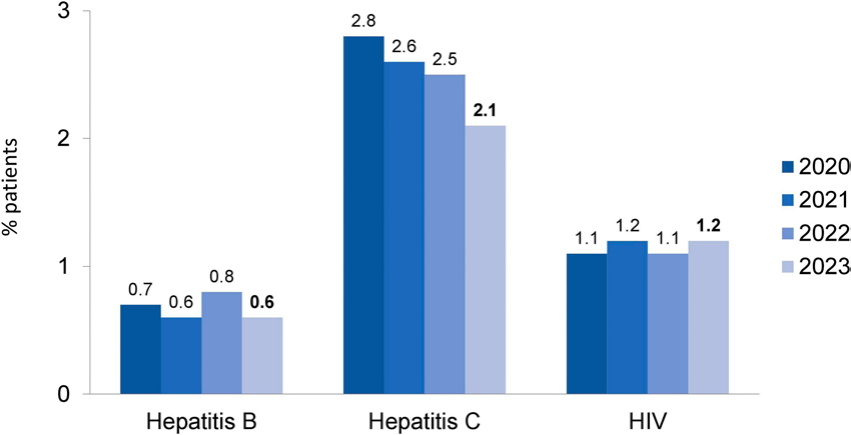
Prevalence of patients with positive serology for hepatitis B and C and HIV.

Fifty-nine percent of patients were males and 41% females. Regarding age, 0.9% were less than 20 years old, 62.5% were between 20 and 64 years, and the remaining 33.6% were older than 65 years. Hypertension was the main underlying disease that led to kidney failure (37%), followed by diabetes mellitus (31%). There was a reduction in glomerulonephritis prevalence (from 8% in 2022 to 5%) (Figure 4). The percentage of patients with positive serology tests for hepatitis C (2.1%; n = 1,345/62,617) and hepatitis B (0.6%; n = 350/62,617) declined, while the percentage for patients with HIV increased slightly (1.2%; n = 747/62,617) (Figure 5).

**Figure 6:**
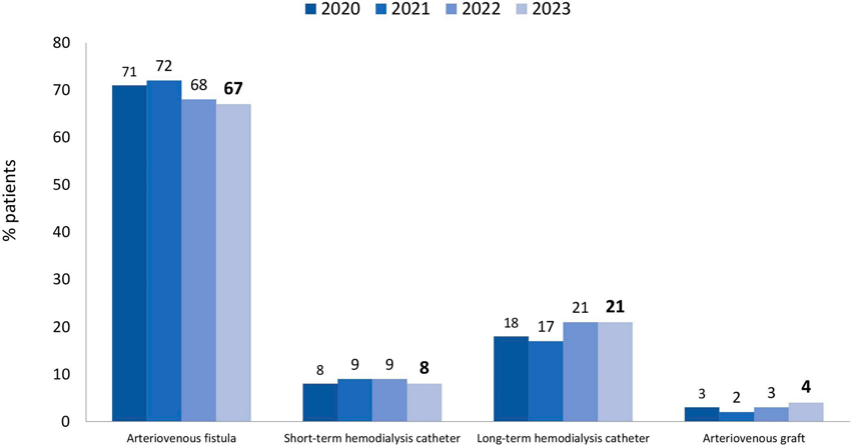
Type of vascular access used for hemodialysis.

**Figure 7:**
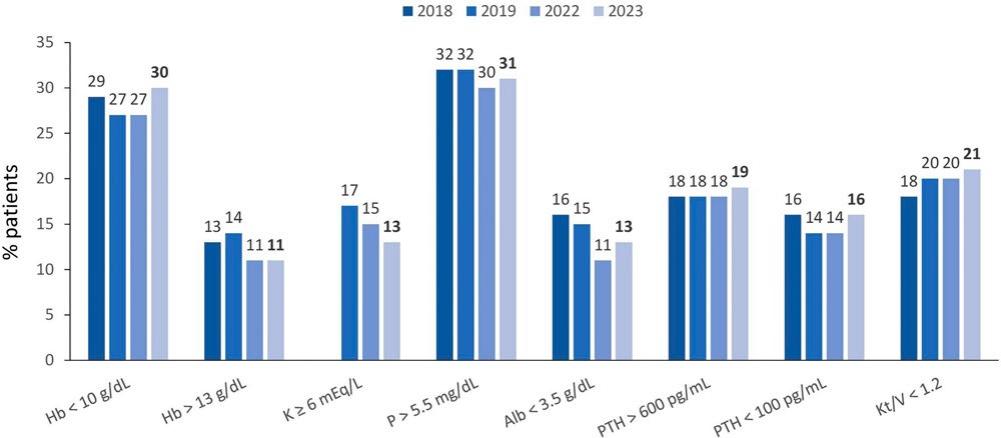
Distribution of dialysis patients according to biochemical results.

**Figure 8:**
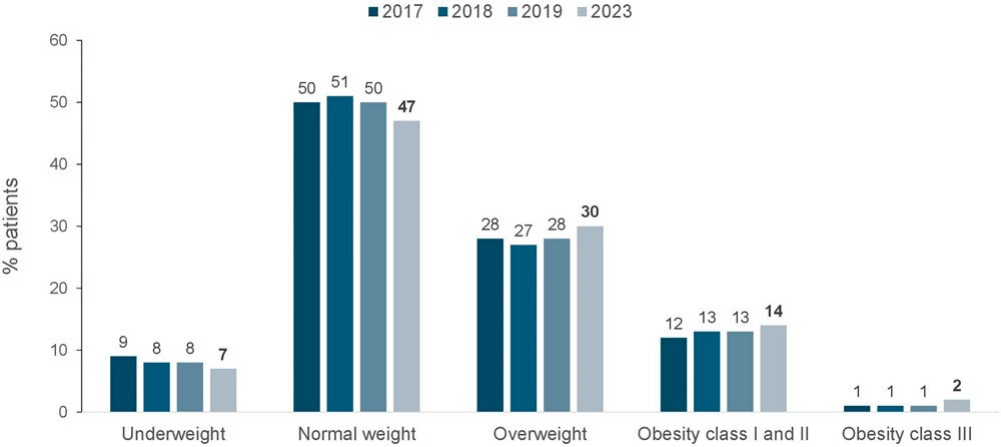
Distribution of dialysis patients according to nutritional status.

**Figure 9:**
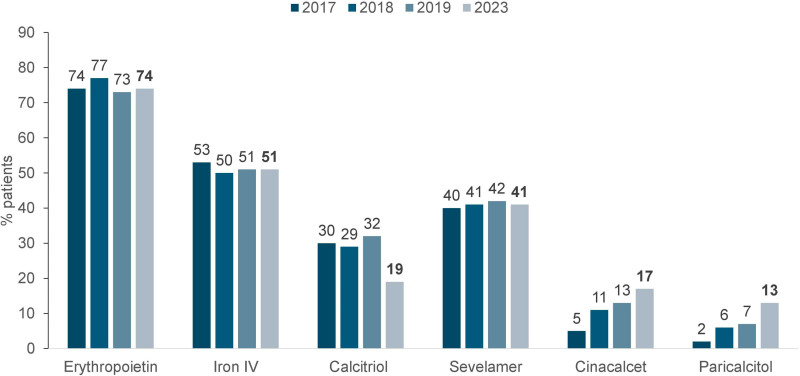
Distribution of dialysis patients according to use of medications.

As to vascular access for hemodialysis or hemodiafiltration, 29% of patients used a central venous catheter (Figure 6). In terms of biochemical parameters compared to the previous year, there was a decrease in the prevalence of patients with serum potassium ≥ 6.0 mEq/L (15 to 13%) and a modest increase in the prevalence of those with hemoglobin <10 g/dL (27 to 30%), while the proportion of those with serum phosphate > 5.5 mg/dL remained stable (30% to 31%) (Figure 7). Compared to the previous year when nutritional status data was collected (2019), the prevalence of overweight or obesity rose from 42 to 46% (Figure 8). The main changes in medication use compared to 2019 were a decrease in calcitriol (from 32% to 19%) and an increase in cinacalcet (from 13% to 17%) and paricalcitol (from 7% to 13%) (Figure 9).

### Characteristics of Dialysis Treatment

**Figure 10:**
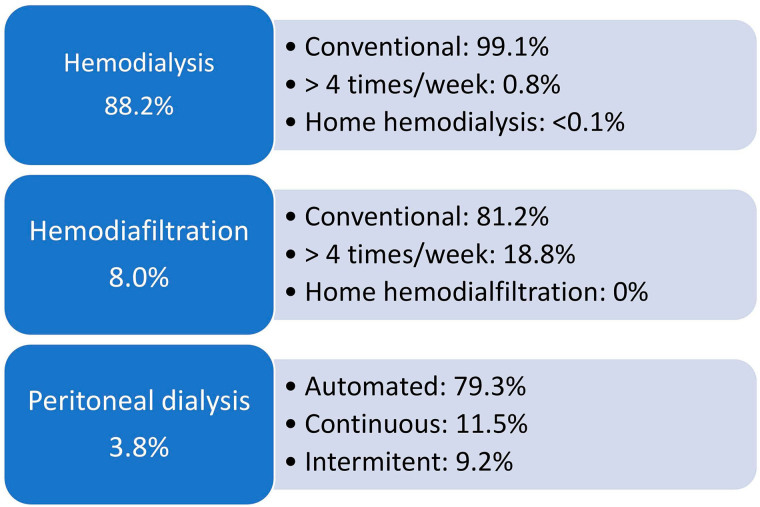
Distribution of patients according to dialysis modality.

The distribution of patients according to the dialysis modality and funding source in the voluntary survey is shown in Table 2. The public health system was the funding source for 76.2% (n = 47,700/62,622) and private health insurance for 23.8% (n = 14,922/62,622). Hemodialysis was the most common dialysis modality (88.2%), followed by hemodiafiltration (8.0%) and peritoneal dialysis (3.8%) (Figure 10). The vast majority (97%) of the patients on hemodiafiltration were funded by the private healthcare system (Table 2). Regarding dialysate characteristics, 78% and 76% of the dialysis facilities used the same bicarbonate concentration and potassium concentration, respectively, for all patients.

**Table 2:** Distribution of patients by modality of dialysis and paying source

Modality	Public health		Private health		Total	
	N	%	N	%	N	%
HD ≤ 4 sessions/week	45858	96.1	8899	59.6	54757	87.4
HD > 4 sessions/week	98	0.2	354	2.4	452	0.7
Home HD	2	0	26	0.2	28	0
HDF ≤ 4 sessions/week	134	0.3	3734	26.4	4068	6.5
HDF > 4 sessions/week	3	0	959	6.4	962	1.5
Home HDF	0	0	0	0	0	0
CAPD	171	0.4	99	0.7	270	0.4
APD	1307	2.7	561	3.8	1868	3
IPD	127	0.3	90	0.6	217	0.3
Total	47700	100	14922	100	62622	100

Abbreviation – HD: hemodialysis; HDF: hemodiafiltration; CAPD: continuous ambulatory peritoneal dialysis; APD: automated peritoneal dialysis; IPD: intermittent peritoneal dialysis.

### Characteristics of Participating Centers

Of the 332 participating dialysis centers, 81% were privately owned, 12% were philanthropic, and 7% were public. Thirty-three percent of the centers were run by international corporations. Most centers were identified as satellite centers (70%), with in-hospital centers comprising 30%. The national mean number of patients per nephrologist, registered nurse, and nurse technician was 27, 38, and 7, respectively.

## Discussion

In this study, we described the main results of the 2023 BDS. The trends observed in recent years were maintained for most variables. Exceptions included a relevant increase in the prevalence of patients with private funding and use of hemodiafiltration as a dialysis modality, a reduced prevalence of glomerulonephritis as a cause of kidney failure, and changes in the use of drugs for chronic kidney disease - mineral bone disorder. The participation rate of dialysis centers in 2023 increased from 28% to 37.5% compared to the previous year^2^.

The estimated prevalence and incidence rates were obtained from a random sample of dialysis facilities and confirmed the upward trend of recent years. Compared to 2022, the total number of patients increased by 2.3%, and the overall prevalence rate from 757 to 771 pmp. This was the first time we employed a national random sample of centers to estimate the prevalence and incidence rates of chronic dialysis therapy in the population. As a result, the current estimates are more reliable since they overcome the biases related to voluntary participation as occurred in previous years. It has been recognized that voluntary participation cannot control for the higher likelihood of including centers more motivated to contribute, with better performance, equipment, organization, and clinical outcomes.

The overall prevalence of people on dialysis (771 pmp) was higher than the average of the 2019 Latin American Society of Nephrology (SLAHN) registry (650 pmp)^3^ and the 2021 European registry (634 pmp)^4^. Conversely, our numbers were substantially lower than those from the United States in 2021 (1,513 pmp)^5^. The 2023 incidence rate (251 pmp) was higher than the 2022 national estimate (214 pmp), higher than in Latin America in 2019 (168 pmp)^3^ and Europe in 2021 (139 pmp)^4^, and lower than in the United States in 2021 (352 pmp)^5^.

The significant increase in the prevalence of dialysis patients funded by the private healthcare system, from ~20% in previous years to 24% in the random sample, which was also found in the voluntary survey, is partly explained by the upward trend in the number of people with private healthcare insurance in Brazil in recent years^6^. Regarding regional differences, although regional income levels partly account for this disparity, they do not fully reflect the national reality. For example, in 2023, 78.6% of the population in the Central-West region and 87.7% in the Northeast region did not have private health insurance^6^.

There was a slight decrease in the crude mortality rate estimate, from 17.1% in 2022 to 16.2% in 2023, a rate lower than the observed in the pre-pandemic years. A reasonable explanation for the finding was the high mortality rate of the most vulnerable dialysis patients, particularly older ones and those with more comorbidities during the pandemic, leaving a healthier survivor population on chronic dialysis.

A different pattern in primary causes of kidney failure was observed, with hypertension increasing from ~32% in the last years to 37% in 2023 and glomerulonephritis decreasing from ~9% to 5%. Although the different pattern observed this year requires further confirmation, it may reflect the pandemic period, when lower access to elective kidney biopsies may have prevented earlier and more precise diagnosis of the etiology of kidney failure. Therefore, many patients who had glomerulonephritis may have been undiagnosed and started dialysis in the post-covid period with an undetermined diagnosis.

The percentage of patients with hepatitis C continued to decrease, reaching 2.1%. The proportional distribution of hemodialysis access types remained stable, with 29% of patients using a central venous catheter. The expertise that nephrologists have developed in recent years in implanting long-term catheters may have contributed to this finding. Almost a third of all patients had anemia (30%) and hyperphosphatemia (31%), and a positive finding was a decrease in the rate of hyperkalemia.

The increase in the prevalence of overweight and obesity, which now affects almost half of patients (46%), follows the trend of the national population^7^, but can also be partly explained by the protective role that obesity plays in the survival of the chronic hemodialysis population, an association also shown in Brazilian patients^8^.

Significant differences were observed in the use of medications to treat hyperparathyroidism, with a decrease in calcitriol and a rise in cinacalcet and paricalcitol. Perhaps the higher availability of these relatively new drugs by the public health system explains these findings.

Regarding dialysis modalities, peritoneal dialysis continued to decline. The model proposed by our public health system, which proves economically unfeasible for most clinics, seems to be the main reason^9^. More patients were undergoing hemodiafiltration in our country. It is conceivable that the higher participation of facilities operated by international corporations this year (33% of all voluntarily participating centers) influenced this result. Moreover, the overrepresentation of centers operated by international corporations may have influenced several parameters resulting in estimates that do not necessarily correspond to the actual national dialysis scenary, an aspect that deserves analysis in forthcoming surveys.

As study limitations, we highlight the estimates made with electronic data collection through voluntary participation, the aggregation of patient data by dialysis centers, the lack of validation of most answers, and the high representation of international corporation facilities in the voluntary survey, which may lead to findings that are more relevant to these units. Since the incidence and prevalence rates were calculated using different sampling methods from previous years, comparisons should be interpreted with caution. As a strength, the random sampling method used to select dialysis centers in our large country with almost 900 active dialysis facilities, improved the validity of prevalence and incidence rates of therapy and the type of dialysis funding estimates.

In conclusion, the 2023 BDS survey confirmed a consistent rise in the prevalence of dialysis patients over the years and the use of hemodiafiltration as a dialysis modality. There was an increase in the use of drugs more recently recommended for the control of hyperparathyroidism.

## Supplementary Material

The following online material is available for this article:

Supplement - Dialysis units participants.
