# Supplementary material for: Brazilian Dialysis Survey 2023
Source: J Bras Nefrol. 2025 Jan 27;47(1):e20240081. doi: 10.1590/2175-8239-JBN-2024-0081en (PMC11801593; doi:10.1590/2175-8239-JBN-2024-0081en)
Supplement: Supplementary file 2 [file 2175-8239-jbn-47-1-e20240081-suppl2.pdf]

Braz. J. Nephrol. (J. Bras. Nefrol.)

https://doi.org/10.1590/2175-8239-JBN-2024-0081pt

Material Suplementar para "Censo Brasileiro de Diálise 2023"

Suplemento - Participantes das unidades de diálise.

RAZÃO SOCIAL	CIDADE	UF
Clínica De Nefrologia De Acailandia	Açailândia	MA
Santa Casa De Misericórdia De Adamantina Na Providência De Deus	Adamantina	SP
Davita Águas Claras Serviços De Nefrologia Ltda.	Águas Claras	DF
Hemovida Serviço De Nefrologia E Hemodiálise Ltda.	Alagoinhas	BA
Casa De Caridade De Alfenas	Alfenas	MG
Cenam - Centro De Nefrologia Amparo	Amparo	SP
Clínica De Hemodiálise Nefrosaùde	Ananindeua	PA
Davita Ananindeua	Ananindeua	PA
Davita Serviços De Nefrologia Ananindeua Ltda	Ananindeua	PA
Martins E Paixão S/S Ltda.	Aparecida de Goiânia	GO
Nefron Ltda	Araçatuba	SP
Instituto De Doenças Renais Do Tocantins	Araguaína	TO
Davita Brasil Participações E Serviços De Nefrologia Ltda. (Filial Arapongas)	Arapongas	SP
Davita Serviços De Nefrologia De Araraquara Ltda.	Araraquara	SP
Centro De Nefrologia De Araripina	Araripina	PE
Davita Serviços De Nefrologia Araruama Ltda.	Araruama	RJ
Clínica De Diálise Araucária Eireli	Araucária	PR
Unidade De Nefrologia De Assis Ltda.	Assis	SP
Biorim S/S	Bacabal	MA
Nefro Rim Sul	Bagé	RS
Fundação Pró-Rim De SC	Balneário Camburiú	SC
Centro De Hemodiálise De Balsas/ Nefrovita	Balsas	MA
Clínica De Doenças Renais De Barbalha Ltda	Barbalha	CE
Medsolution Atividades Medicas Ltda	Barra de São Francisco	ES
Instituto De Tratamento De Doenças Renais E Vasculares Ltda	Barreiras	BA
Serviço De Nefrologia De Barretos S/C Ltda.	Barretos	SP
Hospital Municipal De Barueri Dr. Francisco Moran	Barueri	SP
Davita Bauru Serviços De Nefrologia Ltda.	Bauru	SP
Fundação Para O Desenvolvimento Médico E Hospitalar	Bauru	SP
Hospital Estadual De Bauru	Bauru	SP
IBENE - Instituto Bebedouro De Nefrologia	Bebedouro	SP
Davita Serviços De Nefrologia Belém	BELÉM	PA
Davita Serviços De Nefrologia Marco Ltda	Belém	PA
Davita Serviços De Nefrologia Timbó Ltda	Belém	PA
Dialize (S Campos Serviços Médicos)	Belém	PA

RAZÃO SOCIAL	CIDADE	UF
Fundação Hospital De Clínicas Gaspar Vianna	Belém	PA
Gold Nefro Serviços De Nefrologia Ltda	BELÉM	PA
Instituto Social Mais Saude	Belém	PA
Inbel - Instituto Nefrológico Belford Roxo Ltda	Belford Roxo	RJ
Fresenius Savassi	Belo Horizonte	MG
Fundacao Felice Rosso	BELO HORIZONTE	MG
Associação Renal Vida - Blumenau	Blumenau	SC
Unidade De Diálise Hosp. Das Clínicas De Botucatu - UNESP	Botucatu	SP
Clínica Do Rim Alto Acre	Brasileia	AC
Clínica De Nefrologia Renal Vida Ltda - Me	Brasília	DF
Davita Asa Norte (Antiga Seane)	Brasília	DF
Davita Brasil Participações E Serviços De Nefrologia Ltda. (Filial Asa Norte)	Brasília	DF
Davita Brasil Participações E Serviços De Nefrologia Ltda. (Filial Taguatinga)	Brasília	DF
Davita Ceilândia Serviços De Nefrologia Ltda.	Brasília	DF
Davita Serviços De Nefrologia Asa Sul Ltda.	BRASÍLIA	DF
Davita Serviços De Nefrologia Pacini Ltda.	Brasília	DF
Hospital Universitário HUB-Unb- EBSERH	Brasília	DF
Invictus Nefro Ltda	Brasília	DF
Nephron Brasília Serviços Médicos Ltda - Mix Park	Brasília	DF
Nephron Brasília Serviços Médicos Ltda - Taguatinga	Brasília	DF
Renal Care - Prevenção E Tratamento	Brasília	DF
Nefroclínicas Brasília - Serviços De Nefrologia E Diálise SA	Brasília	DF
Clínica De Diálise Do Cabo	Cabo de Santo Agostinho	PE
Davita Serviços De Nefrologia Cabo Frio Ltda.	Cabo Frio	RJ
Centro De Hemodiálise Da Santa Casa Misericórdia	Cachoeiro de Itapemirim	ES
Hospital Evangélico De Cachoeiro De Itapemirim	Cachoeiro de Itapemirim	ES
Clínica Do Rim Ltda.	Caicó	RN
4health Servicos Medicos Ltda Epp	Caldas Novas	GO
Nefrovida - Centro De Nefrologia E Urologia Da Bahia	Camaçarí	BA
Nefroclínica Ltda.	Camaquã	RS
CTERT Centro De Tratamento Especializado Renal E Transplante Limitada	CAMPINA GRANDE	PB
Sociedade Hospitalar Angelina Caron	Campina Grande do Sul	PR
Davita Serviços De Nefrologia Anchieta Ltda.	Campinas	SP
Davita Serviços De Nefrologia Benjamin Constant Ltda.	Campinas	SP
Davita Serviços De Nefrologia Campinas Ltda.	Campinas	SP
Davita Serviços De Nefrologia Taquaral Ltda	Campinas	SP
Lume Nefrologia E Diálise	CAMPINAS	SP
Davita Serviços De Nefrologia Campo Grande Ltda.	Campo Grande	MS
Med Rim Serviços Médicos Ltda. - Hiper Rim	CAMPO GRANDE	MS
Clínica De Diálise Campo Largo Eireli	Campo Largo	PR
Davita Serviços De Nefrologia Campo Largo Ltda.	Campo Largo	PR
Imne - Instituto De Medicina E Endocrinologia	Campos dos Goytacazes	RJ

RAZÃO SOCIAL	CIDADE	UF
Pró-Rim (Campos)	Campos dos Goytacazes	RJ
Associação Guiomar Jesus De Prevenção E Assistência A Saúde	Capanema	PA
Baxter RCS Centro De Cuidado Renal Ltda - Unidade Cariacica	Cariacica	ES
Medirim	Cariacica	ES
Centro De Hemodiálise Ari Gonçalves LTDA	Castanhal	PA
Casa De Saúde E Maternidade De Caxias	Caxias	MA
Nefroclínica - Clínica De Tratamento Renal	Caxias do Sul	RS
Instituto De Nefrologia De Ceres Ltda.	Ceres	GO
Med Service Serviços Médicos E Gestão Em Saúde Ltda	Chapadinha	MA
Fundação Hospitalar De Saúde	Cianorte	PR
Casa De Saude De Santa Maria S/A	Colatina	ES
Clínica Nefrológica De Colatina Ltda.	Colatina	ES
Clínica De Doenças Renais De Colombo Ltda	Colombo	PR
Nefron Serviços Médicos De Nefrologia Ltda	Contagem	MG
Clínica Nefronor	Cornélio Procópio	PR
Instituto Do Rim Cornélio Procópio	Cornélio Procópio	PR
Hospital Regional De Coxim Dr. Alvaro Fontoura Silva	Coxim	MS
Unidade De Diálise Dr. Raimundo Bezerra - Uni-Rim	Crato	CE
Sociedade Literária E Caritativa Santo Agostinho	Criciúma	SC
Clínica De Tratamento Renal Ltda.	Cuiabá	MT
Clínica Nefrológica De Mato Grosso	CUIABÁ	MT
Davita Cuiabá	Cuiabá	MT
Davita Serviços De Nefrologia Cuiaba Ltda.	Cuiabá	MT
Associação Hospitalar De Proteção À Infância Dr. Raul Carneiro	Curitiba	PR
Associação Hospitalar De Proteção À Infância Dr. Raul Carneiro	Curitiba	PR
Centro De Nefrologia Nações S/S	Curitiba	PR
Clínica De Dialise Cajuru Eireli	Curitiba	PR
Clínica De Doenças Renais Curitiba Eireli (Filial)	Curitiba	PR
Clinica Evangelico Eireli	Curitiba	PR
Davita Serviços De Nefrologia Curitiba Ltda.	Curitiba	PR
Davita Serviços De Nefrologia Vila Izabel Ltda.	Curitiba	PR
Instituto Do Rim Do Paraná	Curitiba	PR
Unirim - Unidade Renal Do Portão Ltda.	Curitiba	PR
Clínica De Hemodiálise De Curitibanos Ltda.	Curitibanos	SC
Unidade Crítica Médica Renal Ltda	Dourados	MS
Cened Centro De Nefrologia De Dourados Ltda	Dourados	MS
Clínica Santa Cruz Ltda.	Eunápolis	BA
Clínica Senhor Do Bonfim Ltda.	Feira de Santana	BA
Irmandade Da Santa Casa De Misericórdia De Fernandópolis	Fernandopolis	SP
Davita Brasil Participações E Serviços De Nefrologia Ltda. (Filial Meireles)	Fortaleza	CE
Davita Brasil Participações E Serviços De Nefrologia Ltda. (Filial Mondubim)	Fortaleza	CE
Davita Brasil Participações E Serviços De Nefrologia Ltda. (Filial São Gerardo)	Fortaleza	CE
Davita Serviços De Nefrologia Meireles LTDA.	Fortaleza	CE
Prontorim Ltda.	Fortaleza	CE

RAZÃO SOCIAL	CIDADE	UF
Prorim Ltda.	Fortaleza	CE
Nefroclínica De Foz Do Iguaçu Ltda.	Foz Do Iguacu	PR
Metta Saúde Ltda.	Foz do Iguaçu	PR
Davita Serviços De Nefrologia Franca Ltda.	Franca	SP
Serviço De Hemodiálise Da Santa Casa De Franca	Franca	SP
Clínica De Doenças Renais Do Sudoeste Ltda.	Francisco Beltrão	PR
Imon Instituto Moratense De Nefrologia Ltda	Francisco Morato	SP
Davita Serviços De Nefrologia Asa Sul Ltda.	GAMA	DF
Casa De Saúde E Maternidade Nossa Senhora Do Perpétuo Socorro	Garanhuns	PE
Clinefro - Clínica De Diálise De Goianésia	Goianésia	GO
Clínica De Doenças Renais	Goiânia	GO
Davita Serviços De Nefrologia Bueno Ltda.	Goiânia	GO
Davita Serviços De Nefrologia Goiania Ltda.	Goiânia	GO
Nefroclínica - Clínica De Doenças Renais Ltda.	Goiânia	GO
Renalclínica - Clínica De Nefrologia Ltda.	Goiânia	GO
Terapia Renal Subst/ Hospital Das Clínicas Da Ufgo	Goiânia	GO
Trs - Terapia Renal Substitutiva	Goiânia	GO
Instituto De Nefrologia Vale Do Rio Doce	Governador Valadares	MG
Servirim - Serviço De Doenças Renais Ltda.	GRAVATAI	RS
Instituto Nefrológico De Guarapari Ltda.	Guarapari	ES
Clire - Clínica De Doenças Renais Ltda.	Guarapuava	PR
Davita Serviços De Nefrologia Guarulhos Ltda.	Guarulhos	SP
Centro Regional De Guaxupé	Guaxupé	MG
Irmandade De Misericórdia De Guaxupé	Guaxupé	MG
Fundação Pró-Rim Gurupi	Gurupi	TO
Davita Serviços De Nefrologia Hortolândia Ltda.	Hortolândia	SP
Clínica De Doenças Renais Imperatriz Ltda.	Imperatriz	MA
Clínica De Nefrologia De Imperatriz	Imperatriz	MA
Clínica Renal Iraty Ltda.	Irati	PR
Centro De Nefrologia Ltda	ITABAIANA	SE
Davita Serviços De Nefrologia Itaboraí Ltda.	Itaboraí	RJ
Centro De Diálise Da Santa Casa De Misericórdia De Itabuna	Itabuna	BA
Associação Renal Vida Itajaí	Itajaí	SC
Centro De Terapia Renal Substitutiva Do Hospital De Clínicas De Itajubá	Itajubá	MG
Clínica Sare - Serviço De Assistência Ao Paciente Renal Ltda -Me	ITAPETINGA	BA
Instituto De Nefrologia E Diálise Itapetininga	Itapetininga	SP
Davita Brasil Participações E Serviços De Nefrologia Ltda. (Filial Itapevi)	Itapevi	SP
Santa Casa De Misericódia De Itatiba	ITATIBA	SP
Santa Casa De Misericordia De Itatiba	Itatiba	SP
Instituto De Hemodiálise De Itumbiara	Itumbiara	GO
Instituto Do Rim De Ivaiporã Ltda.	Ivaiporã	PR
Hospital Do Rim De Janaúba	Janaúba	MG
Centro De Tratamento De Doenças Renais S/C Ltda.	Jaraguá do Sul	SC
Centro De Doenças Renais De Jequié Ltda.	JEQUIÉ	BA

RAZÃO SOCIAL	CIDADE	UF
Centro De Nefrologia E Dialise De Joao Pessoa Ltda	João Pessoa	PB
Davita Serviços De Nefrologia João Pessoa Ltda.	João Pessoa	PB
Fresenius João Pessoa - Centro De Nefrologia E Diálise	João Pessoa	PB
Centro De Tratamento De Doenças Renais SS Ltda.	Joinville	SC
Nefrologia Joinville	Joinville	SC
Fundação Pro Rim- Vida Center	Joinville	SC
Clinefro - Clínica De Nefrologia De Juazeiro Ltda.	Juazeiro	BA
Davita Brasil Participações E Serviços De Nefrologia Ltda. (Filial Juiz De Fora)	Juiz DE Fora	MG
Davita Brasil Participações E Serviços De Nefrologia Ltda. (Filial Rio Branco)	Juiz de Fora	MG
Davita Serviços De Nefrologia Lapa Ltda.	LAPA	SP
Renovare Nefrologia	Leme	SP
Davita Serviços De Nefrologia Linhares Ltda (Filial)	LINHARES	ES
Davita Brasil Participacoes E Servicos De Nefrologia Ltda.	Londrina	PR
Davita Brasil Participações E Serviços De Nefrologia Ltda.	Londrina	PR
Davita Brasil Participações E Serviços De Nefrologia Ltda. (Filial Bandeirantes)	Londrina	PR
Davita Brasil Participações E Serviços De Nefrologia Ltda. (Filial Lago Parque)	Londrina	PR
Davita Brasil Participações E Serviços De Nefrologia Ltda. (Filial Londrina)	LONDRINA	PR
Clínica De Doenças Renais S/A - MACAÉ	Macaé	RJ
Clinica Uninefro Amapa Ltda	Macapá	AP
Centro De Prevenção E Tratamento Das Doenças Renais	Maceió	AL
Clínica Doencas Renais Ltda	Maceió	AL
Santa Casa De Misericórdia De Maceió	Maceió	AL
Centro Tratamento Doenças Renais De Joinville S/C	Mafra	SC
Centro De Nefrologia Mageense Ltda.	Mage	RJ
Centro De Doenças Renais Do Amazonas - CDR	Manaus	AM
Centro De Hemodialise Ari Goncalves Ltda - Epp	MANAUS	AM
Clínica De Doenças Renais E Hipertensão Ltda.	Maracanaú	CE
Instituto Do Rim De Marília	Marília	SP
Associação Beneficente Bom Samaritano	Maringá	PR
Ethos Clin S/S	Maringá	PR
Instituto Do Rim De Maringá S/S LTDA	Maringá	PR
Santa Casa De Misericórdia De Maringá	Maringá	PR
São Francisco Nefrologia. Sociedade Simples	MOGI GUAÇU	SP
Nefroclin - Clínica De Doenças Renais Ltda.	Montenegro	RS
Hospital Do Rim / Irmandade Nossa Senhora Das Mercês	Montes Claros	MG
Hospital Do Rim Ltda	Mossoró	RN
Mossoró Consultória Técnica Em Dialise Ltda	Mossoró	RN
Centro De Nefrologia De Natal	Natal	RN
Davita Natal Serviços De Nefrologia Ltda.	Natal	RN
Davita Serviços De Nefrologia Lagoa Nova Ltda.	NATAL	RN
Instituto Do Rim S/C Ltda	Natal	RN
Nefron Clinica S/A	Natal	RN
Renal Clinica Ltda	Natal	RN
Clínica De Doenças Renais S/A - NITERÓI	Niterói	RJ
Davita Brasil Participações E Serviços De Nefrologia Ltda. (Filial Niterói)	Niterói	RJ

RAZÃO SOCIAL	CIDADE	UF
Centro De Nefrologia De Nova Friburgo	Nova Friburgo	RJ
Clínica De Doenças Renais S/A - Nova Iguaçu	Nova Iguaçu	RJ
Davita Serviços De Nefrologia Nova Iguaçu Ltda.	Nova Iguaçu	RJ
Hospital São João Batista	Nova Prata	RS
Centro Renal - Centro De Prevenção E Tratamento De Doenças Renais Ltda	Novo Hamburgo	RS
Fundação Pró-Rim Palmas	Palmas	TO
Nefro LTDA	Palmas	TO
Santa Casa De Misericórdia De Passos	Passos	MG
Unidade De Terapia Renal De Pato Branco Ltda.	Pato Branco	PR
Unidade De Terapia Renal De Pato Branco Ltda.	Pato Branco	PR
Clínica Do Rim Do Alto Paranaíba	Patos de Minas	MG
Clínica Do Rim - Petrolina	Petrolina	PE
Freire E Ruivo Serviços De Nefrologia	Pindamonhangaba	SP
Clinorte - Clínica De Diálise De Porangatu	Porangatu	GO
Centro De Diálise Do Hospital Moinhos De Vento	Porto Alegre	RS
CND Centro De Nefrologia E Dialise Do Hospital Ernesto Dornelles	Porto Alegre	RS
Hospital Mãe De Deus	Porto Alegre	RS
Hospital Nossa Sra Da Conceicao	Porto Alegre	RS
Instituto De Doenças Renais	Porto Alegre	RS
Instituto De Doenças Renais Ltda.	Porto Alegre	RS
Irmandade Da Santa Casa De Misericórdia De Porto Alegre	Porto Alegre	RS
Vita-Rim Clínica De Doenças Renais Ltda	Porto Alegre	RS
Nefron Serviços De Nefrologia Ltda	Porto Velho	RO
Sos Rim De Porto Velho	Porto Velho	RO
Instituto CEM	Posse	GO
Innefro - Instituto De Nefrologia LTDA	Pouso Alegre	MG
Centro De Nefrologia De Praia Grande	PRAIA	SP
Instituto Do Rim De Presidente Prudente S/C Ltda.	PRESIDENTE PRUDENTE	SP
Davita Brasil Participações E Serviços De Nefrologia Ltda. (Filial Recife)	Recife	PE
Davita Madalena Serviços De Nefrologia Ltda.	Recife	PE
Davita Serviços De Nefrologia Boa Vista Ltda.	Recife	PE
Davita Tejipió Serviços De Nefrologia Ltda.	Recife	PE
Fresenius Ilha Do Leite Ltda	Recife	PE
Hospital Das Clínicas - Serviço De Nefrologia	Recife	PE
Real Hospital Português De Beneficência Em Pernambuco	Recife	PE
Renal Services	Recife	PE
Uninefron - Unidade Nefrológica S. A	Recife	PE
Clínica Nefrologica De Resende Ltda - Epp	Resende	RJ
Hemovida - Clínica De Hemodialise De Ribeira Do Pombal Ltda	Ribeira do Pombal	BA
Hospital Do Rim Acre	Rio Branco	AC
Hospital Do Rim Do Acre	Rio Branco	AC
Clínica De Doenças Renais - Taquara	Rio de Janeiro	RJ
Clínica De Doenças Renais S/A - Vila Da Penha	Rio de Janeiro	RJ
Cnc-Centro Nefrológico De Cascadura	Rio de Janeiro	RJ

RAZÃO SOCIAL	CIDADE	UF
Davita Brasil Participações E Serviços De Nefrologia Ltda. (Filial Botafogo)	Rio de Janeiro	RJ
Davita Rien Serviços De Nefrologia Ltda	Rio de Janeiro	RJ
Davita Serviços De Nefrologia Barra Da Tijuca Ltda.	Rio de Janeiro	RJ
Fresenius Gávea	Rio de Janeiro	RJ
Gamen - Grupo De Assintência Médica Nefrológica	Rio de Janeiro	RJ
Hemodinil-Centro De Hemodiálise E Diagnóstico Eireli	Rio de Janeiro	RJ
Hospital Universitario Pedro Ernesto	Rio de Janeiro	RJ
Pró-Renal Assistência Médica Ltda. – Copacabana	Rio de Janeiro	RJ
Pró-Renal Assistência Médica Ltda. – Tijuca	Rio de Janeiro	RJ
Renalvida - Assistência Integral Ao Renal Ltd	Rio de Janeiro	RJ
Renalvida Assistência Integral Ao Renal Ltda	Rio de Janeiro	RJ
Associação Renal Vida - Rio Do Sul	Rio do Sul	SC
Clínica Hemorim De Rio Verde LTDA	Rio Verde	GO
Clínica Nefrológica De Rio Verde	Rio Verde	GO
Davita Brasil Participações E Serviços De Nefrologia Ltda. (Filial Rolândia)	Rolândia	PR
Sociedade De Protecao A Maternidade E Infancia De Russas	Russas	CE
Clínica Nephron Itapuã	Salvador	BA
Clínica Senhor Do Bonfim Ltda - Csb Rio Vermelho	Salvador	BA
Clínica Senhor Do Bonfim Ltda.	Salvador	BA
Davita Serviços De Nefrologia Fonte Nova Ltda	SALVADOR	BA
Davita Servicos De Nefrologia Salvador Ltda	Salvador	BA
Hospital Ana Neri	Salvador	BA
Hospital São Rafael Sa	Salvador	BA
Ined – Instituto De Nefrologia E Diálise Ltda	Salvador	BA
Centro De Nefrologia Santa Rita Ltda	SANTA CRUZ	RN
UNI-RIM Clínica De Doenças Renais	Santa Cruz do Sul	RS
Politécnica Saúde Ultramed	Santa Maria	DF
Instituto De Nefrologia De Santo Amaro Ltda	Santo Amaro	BA
Davita Brasil Participações E Serviços De Nefrologia Ltda.	Santo André	SP
Davita Brasil Participações E Serviços De Nefrologia Ltda. (Filial Santo André)	Santo André	SP
Davita Transrim Serviços De Nefrologia Ltda.	SANTO ANDRÉ	SP
Instituto Do Rim Do Norte Pioneiro Ltda.	Santo Antônio da Platina	PR
Clinefron - Stoº Antônio De Pádua	Santo Antônio de Pádua	RJ
Davita Brasil Participações E Serviços De Nefrologia Ltda. (Filial Santos)	Santos	SP
Davita Brasil Participações E Serviços De Nefrologia Ltda	Santos	SP
Davita Brasil Participações E Serviços De Nefrologia Ltda. (Filial Monte Serrat)	Santos	SP
Fenix Praiamar - Serviços Médicos Ltda.	Santos	SP
Fundação Pró-Rim	Sao Bento do Sul	SC
Davita Serviços De Nefrologia Silva Jardim Ltda.	São Bernardo do Campo	SP
Davita Brasil Participações E Serviços De Nefrologia Ltda.	São Bernardo do Campo	SP
Davita Brasil Participações E Serviços De Nefrologia Ltda. (Filial São Bernardo)	São Bernardo do Campo	SP
Davita Brasil Participações E Serviços De Nefrologia Ltda.	São Caetano do Sul	SP

RAZÃO SOCIAL	CIDADE	UF
Davita Brasil Participações E Serviços De Nefrologia Ltda. (Filial São Caetano)	São Caetano do Sul	SP
IDR Instituto De Doenças Renais	São João da Boa Vista	SP
Instituto De Doenças Renais - IDR	São João da Boa Vista	SP
Clínica De Doenças Renais Ltda- São João De Meriti	São João de Meriti	RJ
Renalclin - Clínica Doenças Renais Ltda.	São João Del Rei	MG
Renals - Serviço Especializado Em Tratamento Renal	São Joaquim da Barra	SP
Clínica Dialife Sj Rio Preto	São José do Rio Preto	SP
Davita Serviços De Nefrologia São José Do Rio Preto Ltda.	São José do Rio Preto	SP
Fundação Faculdade De Medicina De São José Do Rio Preto	São José do Rio Preto	SP
Davita Serviços De Nefrologia Santos Dumont Ltda.	São José dos Campos	SP
Davita Serviços De Nefrologia Taubaté Ltda. (Filial Sjc)	São José dos Campos	SP
Clínica De Doenças Renais De São José Dos Pinhais	São José dos Pinhais	PR
Santa Casa De Misericórdia De São Lourenço Do Sul	SÃO LOURENÇO DO SUL	RS
Nefroclinicas Sao Luis - Serviço De Nefrologia E Dialise S/A	SÁO LUIS	MA
Centro De Nefrologia Do Maranhão S/C	São Luís	MA
Clínica De Rim E Hipertensão Arterial	São Luís	MA
Hospital De Referência Estadual De Alta Complexidade Dr. Carlos Macieira	São Luís	MA
Instituto Maranhense Do Rim Ltda	São Luís	MA
Serviço De Nefrologia Do Hospital Universitário Do Maranhão - Ufma	São Luís	MA
Unidade De Nefrologia Do Hospital Universitário Do Maranhão	São Luís	MA
Unidade De Terapia Renal Substitutiva De São Mateus Ltda	São Mateus	ES
Clínica Renal Do Extremo Oeste Ltda	São Miguel do Oeste	SC
Clínica De Nefrologia Manoel Villanova Lopes	São Miguel dos Campos	AL
SPDM Sociedade Paulista Para O Desenvolvimento Da Medicina	SAO PAULO	SP
Centro De Diálise Einstein	São Paulo	SP
Clínica E Nefrologia Leste Ltda.	São Paulo	SP
Davita Brasil Participações E Serviços De Nefrologia Ltda.	São Paulo	SP
Davita Brasil Participações E Serviços De Nefrologia Ltda. (Filial Jardim Itapecerica)	São Paulo	SP
Davita Brasil Participações E Serviços De Nefrologia Ltda. (Filial Penha)	São Paulo	SP
Davita Brasil Participações E Serviços De Nefrologia Ltda. (Filial Perdizes)	São Paulo	SP
Davita Serviços De Nefrologia Aricanduva	São Paulo	SP
Davita Serviços De Nefrologia Jardim Das Imbuias Ltda. (Filial Interlagos)	São Paulo	SP
Davita Serviços De Nefrologia Santana Ltda.	São Paulo	SP
Davita Serviços De Nefrologia Vila Olimpia Ltda.	São Paulo	SP
Enesp - Equipe Nefrológica De São Paulo	São Paulo	SP
ENESP (Não Sorteado)	São Paulo	SP
Fenix Analia Franco Serviços Medicos Ltda	SÃO PAULO	SP
Fénix Nefrologia	São Paulo	SP
Fenix Serviços Médicos Ltda	São Paulo	SP
Fresenius Jardins	São Paulo	SP

RAZÃO SOCIAL	CIDADE	UF
Fresenius Jardins- Centro De Nefrologia E Diálise	São Paulo	SP
Fresenius Medical Care Morumbi Ltda	São Paulo	SP
Fresenius Nove De Julho	São Paulo	SP
Fresenius Perdizes	São Paulo	SP
Fresenius Vila Mariana - Centro De Nefrologia E Diálise	São Paulo	SP
Fundação Oswaldo Ramos	São Paulo	SP
Hospital Do Servidor Público Municipal	São Paulo	SP
Nefrocor E Uro Serviços Médicos LTDA	São Paulo	SP
Serviço De Diálise Do Hospital Universitário - USP	São Paulo	SP
Clínica De Nefrologia De Senhor Do Bonfim Ltda - Clinefro	Senhor do Bonfim	BA
Baxter RCS Centro De Cuidado Renal Ltda	Serra	ES
Davita Serviços De Nefrologia Serra Ltda	Serra	ES
Clínica De Nefrologia De Serrinha	Serrinha	BA
Davita Brasil Participações E Serviços De Nefrologia Ltda. (Filial Sobradinho)	Sobradinho	DF
Hospital Regional De Sobradinho	Sobradinho	DF
Santa Casa De Misericórdia/Dialise	Sobral	CE
Clínica Nefrológica Soledade Ltda.	Soledade	RS
Hospital De Caridade Frei Clemente	Soledade	RS
Instituto De Hemodiálise Sorocaba Ltda.	Sorocaba	SP
Secretaria De Saúde De Sp	Sorocaba	SP
Unimed De Sorocaba	Sorocaba	SP
Davita Brasil Participações E Serviços De Nefrologia Ltda. (Filial João Dias)	SP	SP
Davita Serviços De Nefrologia Sumaré Ltda.	Sumaré	SP
Davita Serviços De Nefrologia Taubaté Ltda. (Filial Taubaté)	Taubaté	SP
Saúde Renal Serviços Médicos Ltda	Teixeira de Freitas	BA
Centro De Terapia Renal SS Ltda - CTR - PI	Teresina	PI
Clinefro Nefrologia Ltda	Teresina	PI
Nefrolife	Teresina	PI
Associação Renal Vida - Timbó	Timbó	SC
Centro De Terapia Renal De Timon Ltda.	Timon	MA
Hematol - Clínica De Terapia Renal De Toledo Ltda.	Toledo	PR
Clínica De Doenças Renais De Tubarão S/S Ltda.	Tubarão	SC
Clínica Nefro-Endocrino Ltda Me	Uberlandia	MG
Instituto De Nefrologia Do Triângulo	Uberlândia	MG
Davita Brasil Participações E Serviços De Nefrologia Ltda. (Filial Uber)	Uberlândia	MG
Instituto Do Rim De Umuarama Ltda.	Umuarama	PR
Davita Serviços De Nefrologia Valinhos Ltda.	Valinhos	SP
Da Vita Serviços Médicos Ltda	Valparaíso de Goiás	GO
Davita Brasil Participações E Serviços De Nefrologia Ltda. (Filial Valparaíso)	Valparaíso de Goiás	GO
Servirim Servico De Doencas Renais Ltda - Viamão	Viamão	RS
Clínica De Hemodiálise De Videira	Videira	SC
Associação Evangélica Beneficente Espírito Santense	Vila Velha	ES
Clinirim Clínica Do Rim Ss Ltda	Vila Velha	ES
Instituto Do Rim De Vilhena	Vilhena	RO
Baxter RCS Centro De Cuidado Renal Ltda. - Unidade Vitória	Vitória	ES

RAZÃO SOCIAL	CIDADE	UF
Davita Serviços De Nefrologia Vitória Ltda	Vitória	ES
Hospital Santa Rita De Cássia	Vitória	ES
Hospital Universitário Cassiano Antônio Moraes - Hucam	Vitória	ES
Cdvr - Clínica De Diálise De Volta Redonda	Volta Redonda	RJ
Instituto Do Rim	Votuporanga	SP
