# Supplementary material for: Brazilian Dialysis Survey 2023
Source: J Bras Nefrol. 2025 Jan 27;47(1):e20240081. doi: 10.1590/2175-8239-JBN-2024-0081en (PMC11801593; doi:10.1590/2175-8239-JBN-2024-0081en)
Supplement: Translation

# Translation: Censo Brasileiro de Diálise 2023

**DOI:** 10.1590/2175-8239-JBN-2024-0081pt

**Authors:** Fabiana B. Nerbass, Helbert do Nascimento Lima, Jorge P. Strogoff-de-Matos, Bruno Zawadzki, José A. Moura-Neto, Jocemir Ronaldo Lugon, Ricardo Sesso

**Affiliations:** 1 Fundação Pró-Rim, Joinville, SC, Brasil.; 2 Universidade da Região de Joinville, Joinville, SC, Brasil.; 3 Fresenius Medical Care, Rio de Janeiro, Brasil.; 4 DaVita Tratamento Renal, Rio de Janeiro, RJ, Brasil.; 5 Escola Bahiana de Medicina e Saúde Pública, Salvador, BA, Brasil.; 6 Universidade Federal Fluminense, Niterói, RJ, Brasil.; 7 Universidade Federal de São Paulo, São Paulo, SP, Brasil.

**Keywords:** Hemodiálise, Diálise Peritoneal, Epidemiologia

## Abstract

**Introdução:**

O Censo Brasileiro de Diálise (CBD) anual apoia e contribui para o desenvolvimento de políticas nacionais de saúde.

**Objetivo:**

Reportar dados epidemiológicos de 2023 do CBD da Sociedade Brasileira de Nefrologia (SBN).

**Métodos:**

Realizou-se pesquisa em amostra voluntária de centros brasileiros de diálise utilizando questionário online, abrangendo aspectos clínicos e epidemiológicos de pacientes em diálise crônica e características dos centros. Para estimativas de prevalência, incidência e fonte de financiamento da diálise, obtivemos dados de uma amostra aleatória de centros de diálise.

**Resultados:**

Um total de 37,5% (n = 332) dos centros respondeu voluntariamente ao questionário online; 124 foram selecionados aleatoriamente para estimativas específicas de taxas de prevalência e incidência. Estimou-se que, em 1º de julho de 2023, o número total de pacientes em diálise era 157.357 e 51.153 iniciaram a diálise em 2023. As taxas estimadas de prevalência e incidência de pacientes por milhão da população (pmp) foram de 771 e 251, respectivamente. Dos pacientes prevalentes, 88,2% estavam em hemodiálise, 8,0% em hemodialfiltração e 3,8% em diálise peritoneal. A prevalência de anemia (Hb <10g/dL) foi 29% e de hiperfosfatemia (P > 5,5mg/dL), 30%. Houve aumento na frequência do uso de cinacalcete e paricalcitol. A taxa bruta global de mortalidade anual estimada foi 16,2%.

**Conclusões:**

Estimativas de uma amostra aleatória de centros de diálise mostram que o número absoluto e a taxa de prevalência de pacientes em diálise crônica continuam a aumentar. Um número crescente de pacientes foi submetido à hemodiafiltração e ao uso de cinacalcete e paricalcitol para tratamento de hiperparatireoidismo.

## Introdução

Desde 1999, a Sociedade Brasileira de Nefrologia (SBN) promove o Censo Brasileiro de Diálise (CBD), uma pesquisa nacional online com o objetivo de coletar e analisar aspectos epidemiológicos e clínicos de pacientes submetidos à diálise crônica. Essa iniciativa fornece informações relevantes para o desenvolvimento de políticas de saúde e estratégias para melhorar o cuidado de indivíduos em tratamento de diálise crônica no país.

Pesquisas anteriores utilizaram somente amostras de participantes voluntários, o que pode ter tornado os resultados mais suscetíveis a vieses de seleção. Neste ano, além da participação voluntária para obtenção de parte dos dados, pela primeira vez, também foram coletados dados de uma amostra aleatória de centros de diálise em todo o país, a fim de aumentar a precisão das estimativas das taxas de prevalência, taxas de incidência e fonte de financiamento para o tratamento dialítico.

Este artigo apresenta os principais resultados do CBD 2023 e os compara com pesquisas anteriores.

## Métodos

### Prevalência, Incidência e Fonte de Financiamento da Diálise

Para estimativas de prevalência, incidência e fonte de financiamento para diálise, os dados foram obtidos através de uma amostragem aleatória de centros de diálise. A partir de uma lista de centros em cada região geográfica (Norte, Nordeste, Centro-Oeste, Sudeste e Sul), as instalações foram selecionadas por meio de um método de amostragem aleatória sequencial. A unidade de diálise inicial foi selecionada com base em uma tabela de números aleatórios e as unidades subsequentes seguiram uma sequência fixa de repetições. Foram selecionados aproximadamente 20% dos centros de diálise em cada região geográfica, à exceção do Sudeste, que conta com um total de 450 centros ativos, dos quais foi sorteada uma amostra de 15%. Para compensar a falta de resposta, foi incluído um adicional de 50% dos centros selecionados. Uma meta mínima de 70% do número planejado de centros de diálise foi estabelecida.

Inicialmente, as unidades de diálise selecionadas foram contatadas por e-mail para informar os seguintes dados: 1. Número total de pacientes em diálise crônica em 1º de julho de 2023; 2. Número de pacientes com financiamento de diálise por plano de saúde privado em 1º de julho de 2023; e 3. Número de pacientes que iniciaram diálise crônica durante o mês de julho de 2023. Para reforçar o percentual de respondentes, foi realizado um contato telefônico subsequente com as unidades que não responderam às mensagens eletrônicas.

Para o cálculo das estimativas, as regiões do país (Norte, Nordeste, Centro-Oeste, Sudeste e Sul) foram consideradas como estratos, e observou-se que o compartilhamento de amostras entre os estratos não foi proporcional. Como resultado, foram introduzidos pesos na etapa de análise dos dados para compensar as variações nas probabilidades de amostragem e restaurar a proporcionalidade (Tabela 1). Como as taxas de resposta variaram por região, os pesos foram ajustados para permitir a distribuição da amostra entre os estratos em relação à população do estudo. Dado o método de amostragem empregado, amostragem estratificada simples, estimou-se o número médio de pacientes em diálise por centro em 1º de julho e calculou-se o número médio de novos pacientes no ano para cada região do país, com os respectivos intervalos de confiança de 95% (IC 95%). Em seguida, os resultados foram expandidos para o número total de unidades em cada região. O percentual de pacientes financiados por planos de saúde públicos e privados foi obtido por meio de proporção simples na amostra selecionada.

**Tabela 1:** Procedimentos de amostragem em instalações de diálise em todo o país, frações de amostragem, pesos do desenho, taxas de resposta e pesos ajustados por região

Região	Instalações			Fração de amostragem	Peso do desenho	Taxa de resposta	Peso ajustado
	Existentes	Amostragem	Respondentes				
Norte	54	18	10	0,333	3,003	0,556	5,401
Nordeste	167	51	27	0,305	3,279	0,529	6,198
Centro-Oeste	87	27	17	0,310	3,226	0,630	5,120
Sudeste	421	95	40	0,225	4,444	0,421	10,555
Sul	157	48	30	0,305	3,278	0,625	5,244
Total	886	239	124			0,5188	

Notas – Fração de amostragem = centros de amostragem/existentes; Peso do desenho = 1/fração de amostragem; Taxa de resposta = centros respondentes/amostragem; Peso ajustado = peso do desenho/taxa de resposta

### Dados da Pesquisa Voluntária

As pessoas designadas pelos centros de diálise que responderam à pesquisa preencheram um questionário online no site da SBN. O questionário continha perguntas sobre os parâmetros sociodemográficos, clínicos e terapêuticos dos pacientes em diálise crônica e esteve disponível de agosto a novembro de 2023. A participação na pesquisa foi voluntária e todos os centros de diálise registrados na SBN foram convidados a participar por e-mail, bem como pelos meios de comunicação da SBN. Após o convite inicial, novos lembretes foram enviados mensalmente aos centros que ainda não haviam fornecido seus dados, e foi feito contato direto com os presidentes regionais da SBN e gerentes de corporações internacionais que oferecem serviços de diálise no Brasil.

Os dados de cada centro foram coletados de forma agregada, em vez de individualmente (questionou-se a prevalência de características predeterminadas, como o número de pacientes em hemodiálise, o número de pacientes com doença renal por diabetes, o número de pacientes do sexo masculino e outras características relacionadas à terapia renal substitutiva). A mortalidade anual foi estimada a partir de eventos ocorridos em julho de 2023. Para calcular as taxas de prevalência e incidência, utilizamos dados populacionais nacionais e regionais do Instituto Brasileiro de Geografia e Estatística (IBGE): população total do censo de 2022 mais a taxa média de crescimento anual de 0,52%^1^.

A maioria dos dados foi descritiva e os resultados foram comparados com os dados de anos anteriores.

O número total anual estimado de óbitos foi obtido da seguinte forma: o número de óbitos registrados em julho multiplicado por 12, dividido pela proporção de centros participantes ativos.

A taxa bruta de mortalidade anual estimada (%) foi obtida com base em: o número total estimado de óbitos em 2022 multiplicado por 100, dividido pelo número estimado de pacientes em diálise em 1º de julho.

## Resultados

Em julho de 2023, foram registrados na SBN 886 centros de diálise crônica ativos, um aumento de 1,6% em relação a 2022. Em todo o país, havia 4,3 centros de diálise por milhão da população (pmp), com taxas mais baixas nas regiões Nordeste (3,0 pmp) e Norte (3,1 pmp) em comparação com as regiões Sudeste (4,9 pmp), Centro-Oeste (5,3 pmp) e Sul (5,2 pmp).

### Estimativas Da Amostra Aleatória

#### Taxas estimadas de incidência e prevalência a partir da amostra aleatória

Quatorze por cento dos centros de diálise ativos no Brasil (124/886) participaram da amostra aleatória, 18% dos centros da região Norte (10/54), 16% da região Nordeste (27/167), 19% da região Centro-Oeste (17/87), 9% da região Sudeste (40/421) e 19% da região Sul (30/157).

O número total estimado de pacientes em 1º de julho de 2023 foi de 157.357 (IC 95% 139.164–175.153), 2,3% superior ao de julho de 2022, confirmando a tendência de aumento no número de pacientes em diálise observada nos últimos anos (Figura 1). A taxa de prevalência de pacientes em diálise também continuou a aumentar, de 758 pmp em 2022 para 771 pmp (IC 95% 682–858 pmp) em 2023. Ao analisar esse indicador por região, observa-se uma redução nas taxas do Norte e do Sul (Figura 2). O número estimado de novos pacientes em diálise em 2023 foi de 51.153. A taxa de incidência geral foi de 251 pmp (IC 95% 213–288 pmp), superior à de 2022, quando atingiu 214 pmp, variando de 137 pmp no Norte a 311 pmp no Centro-Oeste.

**Figura 1:** Número estimado de pacientes em diálise crônica por ano.

**Figura 2:** Taxa de prevalência estimada de pacientes em diálise por região geográfica no Brasil, por milhão da população.

A distribuição dos pacientes de acordo com as fontes de financiamento observada na pesquisa de amostragem aleatória foi de 75,6% financiados pelo sistema público de saúde e 24,4% por planos de saúde privados. O financiamento público variou entre as regiões, com maior participação no Nordeste (84%), seguido pelo Sul (81%), Norte (76%), Sudeste (70%) e Centro-Oeste (64%).

### Pesquisa Voluntária

A pesquisa voluntária contou com 332 (37,5%) centros participantes, uma porcentagem maior do que nos últimos três anos, que foi de cerca de 30%. Ao considerar a taxa de resposta pelo número de centros de diálise por região, a região com maior participação foi a Centro-Oeste (46%), seguida pela região Nordeste (41%), Sul (39%), Sudeste (34%) e Norte (33%). O número de pacientes no atual CBD foi 46% maior do que em 2022 (62.617 *vs.* 42.868).

O número estimado de óbitos em todo o ano foi de 27.142. A taxa bruta de mortalidade anual apresentou uma leve redução, passando de 17,1% em 2022 para 16,2% em 2023 (Figura 3).

**Figura 3:** Taxa bruta de mortalidade anual estimada de pacientes em diálise.

### Características Demográficas e Clínicas

Cinquenta e nove por cento dos pacientes eram do sexo masculino e 41% do sexo feminino. Em relação à idade, 0,9% tinham menos de 20 anos, 62,5% tinham entre 20 e 64 anos e os 33,6% restantes tinham mais de 65 anos. Hipertensão foi a principal doença subjacente que levou à doença renal crônica (37%), seguida por diabetes mellitus (31%). Houve uma redução na prevalência de glomerulonefrite (de 8% em 2022 para 5%) (Figura 4). O percentual de pacientes com sorologia positiva para hepatite C (2,1%; n = 1.345/62.617) e hepatite B (0,6%; n = 350/62.617) diminuiu, enquanto o percentual de pacientes com HIV aumentou ligeiramente (1,2%; n = 747/62.617) (Figura 5).

**Figura 4:** Distribuição dos pacientes em diálise de acordo com a etiologia da doença renal crônica.

**Figura 5:** Prevalência de pacientes com sorologia positiva para hepatite B e C e HIV.

Com relação ao acesso vascular para hemodiálise ou hemodiafiltração, 29% dos pacientes utilizavam cateter venoso central (Figura 6). Quanto aos parâmetros bioquímicos, em comparação com o ano anterior, houve uma redução na prevalência de pacientes com potássio sérico ≥ 6,0 mEq/L (15 para 13%) e um aumento discreto na prevalência de pacientes com hemoglobina < 10 g/dL (27 para 30%), enquanto a proporção de pacientes com fósforo sérico > 5,5 mg/dL permaneceu estável (30% para 31%) (Figura 7). Em comparação com o último ano em que foram coletados dados sobre o estado nutricional (2019), a prevalência de sobrepeso ou obesidade aumentou de 42 para 46% (Figura 8). As principais alterações no uso de medicamentos em relação a 2019 foram uma redução no uso de calcitriol (de 32% para 19%) e um aumento em cinacalcete (de 13% para 17%) e paricalcitol (de 7% para 13%) (Figura 9).

**Figura 6:** Tipo de acesso vascular usado para hemodiálise.

**Figura 7:** Distribuição dos pacientes em diálise de acordo com os resultados bioquímicos.

**Figura 8:** Distribuição dos pacientes em diálise de acordo com o estado nutricional.

**Figura 9:** Distribuição dos pacientes em diálise de acordo com o uso de medicamentos.

### Características do Tratamento de Diálise

A distribuição de pacientes de acordo com a modalidade de diálise e a fonte de financiamento na pesquisa voluntária é apresentada na Tabela 2. O sistema público de saúde foi a fonte de financiamento para 76,2% (n = 47.700/62.622) enquanto os planos de saúde privados foram responsáveis por 23,8% (n = 14.922/62.622). A hemodiálise foi a modalidade de diálise mais comum (88,2%), seguida por hemodiafiltração (8,0%) e diálise peritoneal (3,8%) (Figura 10). A grande maioria (97%) dos pacientes em hemodiafiltração foi financiada pelo sistema de saúde privado (Tabela 2). Com relação às características do dialisato, 78% e 76% das unidades de diálise utilizavam a mesma concentração de bicarbonato e de potássio, respectivamente, para todos os pacientes.

**Tabela 2:** Distribuição de pacientes por modalidade de diálise e fonte de financiamento

Modalidade	Saúde pública		Saúde privada		Total	
	N	%	N	%	N	%
HD ≤ 4 sessões/semana	45858	96,1	8899	59,6	54757	87,4
HD > 4 sessões/semana	98	0,2	354	2,4	452	0,7
HD domiciliar	2	0	26	0,2	28	0
HDF ≤ 4 sessões/semana	134	0,3	3734	26,4	4068	6,5
HDF > 4 sessões/semana	3	0	959	6,4	962	1,5
HDF domiciliar	0	0	0	0	0	0
DPAC	171	0,4	99	0,7	270	0,4
DPA	1307	2,7	561	3,8	1868	3
DPI	127	0,3	90	0,6	217	0,3
Total	47700	100	14922	100	62622	100

Abreviaturas – HD: hemodiálise; HDF: hemodiafiltração; DPAC: diálise peritoneal ambulatorial contínua; DPA: diálise peritoneal automatizada; DPI: diálise peritoneal intermitente.

**Figura 10:** Distribuição dos pacientes de acordo com a modalidade de diálise.

### Características dos Centros Participantes

Dos 332 centros de diálise participantes, 81% eram de propriedade privada, 12% eram filantrópicos e 7% eram públicos. Trinta e três por cento dos centros eram administrados por empresas internacionais. A maioria dos centros foi identificada como centros satélites (70%), e os centros hospitalares corresponderam a 30%. O número médio nacional de pacientes por nefrologista, enfermeiro e técnico de enfermagem foi de 27, 38 e 7, respectivamente.

## Discussão

Neste estudo, descrevemos os principais resultados do CBD 2023. As tendências observadas nos últimos anos foram mantidas para a maioria das variáveis. As exceções incluíram um aumento na prevalência de pacientes com financiamento privado e uso de hemodiafiltração como modalidade de diálise, uma redução na prevalência de glomerulonefrite como causa de doença renal crônica e alterações no uso de medicamentos para distúrbio mineral ósseo. Em comparação com o ano anterior, a taxa de participação dos centros de diálise em 2023 aumentou de 28% para 37,5%^2^.

As taxas estimadas de prevalência e incidência foram obtidas a partir de uma amostra aleatória de unidades de diálise e confirmaram a tendência de aumento dos últimos anos. Em comparação com 2022, o número total de pacientes aumentou em 2,3%, e a taxa de prevalência geral de 757 para 771 pmp. Essa foi a primeira vez que empregamos uma amostra aleatória de centros para estimar as taxas de prevalência e incidência da terapia de diálise crônica na população. Como resultado, as estimativas atuais são mais confiáveis, pois superam os vieses relacionados à participação voluntária, como ocorreu em anos anteriores. Reconheceu-se que a participação voluntária não pode controlar a maior probabilidade de inclusão de centros mais motivados em contribuir, são os com melhor desempenho, equipamentos, organização e desfechos clínicos.

A prevalência geral de pessoas em diálise (771 pmp) foi superior à média do registro da Sociedade Latino-Americana de Nefrologia e Hipertensão (SLANH) de 2019 (650 pmp)^3^ e do registro europeu de 2021 (634 pmp)^4^. Em contrapartida, nossos números foram substancialmente inferiores aos dos Estados Unidos em 2021 (1.513 pmp)^5^. A taxa de incidência de 2023 (251 pmp) foi maior do que a estimativa nacional de 2022 (214 pmp), superior à registrada na América Latina em 2019 (168 pmp)^3^ e na Europa em 2021 (139 pmp)^4^ e inferior à dos Estados Unidos em 2021 (352 pmp)^5^.

O aumento significativo na prevalência de pacientes em diálise financiados pelo sistema de saúde privado, de ~20% em anos anteriores para 24% na amostra aleatória, que também foi encontrado na pesquisa voluntária, é parcialmente explicado pela tendência de crescimento no número de pessoas com planos de saúde privados no Brasil nos últimos anos^6^. Em relação às diferenças regionais, embora os níveis de renda regionais sejam parcialmente responsáveis por essa disparidade, eles não refletem totalmente a realidade nacional. Por exemplo, em 2023, 78,6% da população da região Centro-Oeste e 87,7% da região Nordeste não possuíam plano de saúde privado^6^.

Houve uma ligeira redução na estimativa da taxa bruta de mortalidade, de 17,1% em 2022 para 16,2% em 2023, uma taxa inferior à observada nos anos pré-pandemia. Uma explicação razoável para o achado foi a elevada taxa de mortalidade dos pacientes em diálise mais vulneráveis durante a pandemia, especialmente os mais idosos e aqueles com mais comorbidades, resultando em uma população de sobreviventes mais saudável em diálise crônica.

Foi observado um padrão diferente nas causas primárias de insuficiência renal, com a hipertensão aumentando de ~32% nos últimos anos para 37% em 2023 e a glomerulonefrite sofrendo uma redução de ~9% para 5%. Embora o padrão diferente observado neste ano requeira confirmação adicional, ele pode refletir o período pandêmico, quando o menor acesso a biópsias renais eletivas pode ter impedido um diagnóstico mais precoce e preciso da etiologia da doença renal. Portanto, muitos pacientes que apresentaram glomerulonefrite podem ter ficado sem diagnóstico e iniciado a diálise no período pós-covid com um diagnóstico indeterminado.

O percentual de pacientes com hepatite C continuou a diminuir, chegando a 2,1%. A distribuição proporcional dos tipos de acesso para hemodiálise permaneceu estável, com 29% dos pacientes utilizando cateter venoso central. O conhecimento especializado que os nefrologistas desenvolveram nos últimos anos na implantação de cateteres de longa permanência pode ter contribuído para esse achado. Quase um terço de todos os pacientes apresentou anemia (30%) e hiperfosfatemia (31%), e um achado positivo foi a diminuição da taxa de hipercalemia.

O aumento na prevalência de sobrepeso e obesidade, que agora está presente em quase metade dos pacientes (46%), segue a tendência da população nacional^7^, mas também pode ser parcialmente explicado pelo papel protetor que a obesidade desempenha na sobrevida da população em hemodiálise crônica, uma associação também demonstrada em pacientes brasileiros^8^.

Foram observadas diferenças significativas no uso de medicamentos para tratar o hiperparatireoidismo, com uma redução no uso de calcitriol e um aumento no uso de cinacalcete e paricalcitol. Talvez a maior disponibilidade desses medicamentos relativamente novos pelo sistema público de saúde explique esses achados.

Com relação às modalidades de diálise, a diálise peritoneal continuou em declínio. O modelo proposto pelo nosso sistema público de saúde, que se mostra economicamente inviável para a maioria das clínicas, parece ser a principal razão^9^. Mais pacientes foram submetidos à hemodiafiltração em nosso país. É possível que a maior participação de instalações operadas por corporações internacionais neste ano (33% de todos os centros que participaram voluntariamente) tenha influenciado esse resultado. Além disso, a super-representação de centros operados por empresas internacionais pode ter influenciado diversos parâmetros, resultando em estimativas que não necessariamente correspondem ao real cenário nacional da diálise, um aspecto que merece ser analisado em pesquisas futuras.

Como limitações do estudo, destacamos as estimativas realizadas com coleta eletrônica de dados por meio de participação voluntária, a agregação de dados de pacientes por centros de diálise, a falta de validação das respostas e a alta representação de unidades de empresas internacionais na pesquisa voluntária, o que pode levar a achados mais relevantes para essas unidades. Uma vez que as taxas de incidência e prevalência foram calculadas por meio de métodos de amostragem diferentes dos anos anteriores, as comparações devem ser interpretadas com cautela. Como ponto forte, o método de amostragem aleatória usado para selecionar centros de diálise em nosso extenso país, com quase 900 instalações de diálise ativas, melhorou a validade das taxas de prevalência e incidência da terapia e as estimativas do tipo de financiamento da diálise.

Em conclusão, o levantamento do CBD de 2023 confirmou um aumento consistente na prevalência de pacientes em diálise ao longo dos anos e o uso de hemodiafiltração como modalidade de diálise. Houve um aumento no uso de medicamentos mais recentemente recomendados para o controle do hiperparatireoidismo.

## Material Suplementar

O seguinte material online está disponível para este artigo:

Suplemento - Participantes das unidades de diálise.
